# Improved postprocessing of dynamic glucose-enhanced CEST MRI for imaging brain metastases at 3 T

**DOI:** 10.1186/s41747-023-00390-5

**Published:** 2023-12-08

**Authors:** Yulun Wu, Sophie H. A. E. Derks, Tobias C. Wood, Erik de Blois, Astrid A. M. van der Veldt, Marion Smits, Esther A. H. Warnert

**Affiliations:** 1 Department of Radiology & Nuclear Medicine, Erasmus MC, Rotterdam, Netherlands; 2 Brain Tumor Centre, Erasmus MC Cancer Institute, Rotterdam, Netherlands; 3 Departments of Neurology, Erasmus MC, Rotterdam, Netherlands; 4 Departments of Medical Oncology, Erasmus MC, Rotterdam, Netherlands; 5 Department of Neuroimaging, Institute of Psychiatry, Psychology & Neuroscience, King’s College London, London, UK; 6 Medical Delta, Delft, Netherlands

**Keywords:** Brain neoplasms, Glucose, Magnetic resonance imaging, Positron-emission tomography, Principal component analysis

## Abstract

**Background:**

Dynamic glucose-enhanced (DGE) chemical exchange saturation transfer (CEST) has the potential to characterize glucose metabolism in brain metastases. Since the effect size of DGE CEST is small at 3 T (< 1%), measurements of signal-to-noise ratios are challenging. To improve DGE detection, we developed an acquisition pipeline and extended image analysis for DGE CEST on a hybrid 3-T positron emission tomography/magnetic resonance imaging system.

**Methods:**

This cross-sectional study was conducted after local ethical approval. Static Z-spectra (from -100 to 100 ppm) were acquired to compare the use of 1.2 *versus* 2 ppm to calculate static glucose-enhanced (glucoCEST) maps in 10 healthy volunteers before and after glucose infusion. Dynamic CEST images were acquired during glucose infusion. Image analysis was optimized using motion correction, dynamic *B*_0_ correction, and principal component analysis (PCA) to improve the detection of DGE CEST in the sagittal sinus, cerebrospinal fluid, and grey and white matter. The developed DGE CEST pipeline was applied to four patients diagnosed with brain metastases.

**Results:**

GlucoCEST was strongest in healthy tissues at 2 ppm. Correcting for motion, *B*_0,_ and use of PCA locally improved DGE maps. A larger contrast between healthy tissues and enhancing regions in brain metastases was found when dynamic *B*_0_ correction and PCA denoising were applied.

**Conclusion:**

We demonstrated the feasibility of DGE CEST with our developed acquisition and analysis pipeline at 3 T in patients with brain metastases. This work enables a direct comparison of DGE CEST to 18F-fluoro-deoxy-D-glucose positron emission tomography of glucose metabolism in patients with brain metastases.

**Relevance statement:**

Contrast between brain metastasis and healthy brain tissue in DGE CEST MR images is improved by including principle component analysis and dynamic magnetic field correction during postprocessing. This approach enables the detection of increased DGE CEST signal in brain metastasis, if present.

**Key points:**

• Despite the low signal-to-noise ratio, dynamic glucose-enhanced CEST MRI is feasible at 3 T.

• Principal component analyses and dynamic magnetic field correction improve DGE CEST MRI.

• DGE CEST MRI does not consequently show changes in brain metastases compared to healthy brain tissue.

• Increased DGE CEST MRI in brain metastases, if present, shows overlap with contrast enhancement on T1-weighted images.

**Graphical Abstract:**

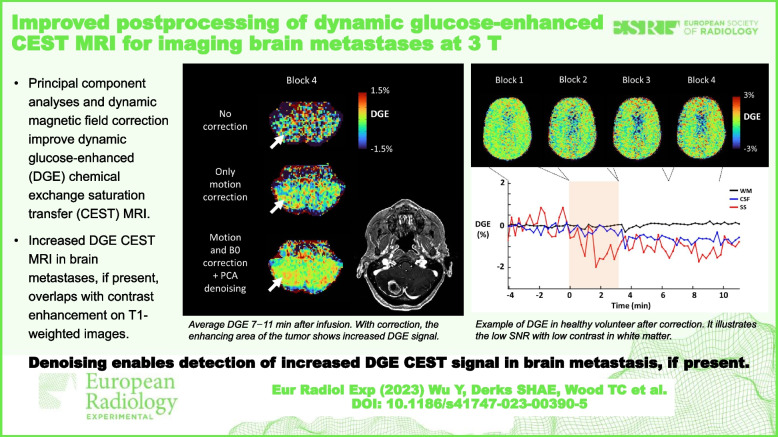

**Supplementary Information:**

The online version contains supplementary material available at 10.1186/s41747-023-00390-5.

## Background

Most malignant neoplasms have an increased rate of glycolysis and glucose uptake, known as the Warburg effect [1]. This is the basis for the extensive use of 18F-fluoro-deoxy-D-glucose for positron emission tomography (PET) in oncology. Chemical exchange saturation transfer (CEST) is a magnetic resonance imaging (MRI) technique that detects the signal of exchangeable protons, such as in the hydroxyl groups of glucose. For CEST, both static [2] and dynamic scanning [3, 4] have been applied. In brain tumors, previous studies have shown that glucose has potential as a contrast agent of CEST [5–8]. In a preclinical study with human glioma xenografts, dynamic glucose-enhanced (DGE) CEST, in which an intravenous bolus injection of D-glucose is measured over time, was able to detect increased blood–brain barrier permeability in tumors [9]. In addition, Xu et al. [10] assessed DGE-CEST at 7 T in patients with glioma and detected CEST signal changes in the brain and glioma following intravenous glucose infusion, thereby demonstrating the feasibility of DGE-CEST in patients with brain tumors. Note that in this paper, we use glucoCEST to refer to glucose enhancement detected by static scans, and “DGE” to refer to glucose-enhanced signal detected by dynamic scans, to be in line with the recent comprehensive review written by Knutsson et al. [11].

To evaluate the feasibility of glucose-enhanced CEST imaging for clinical practice, subsequent research focused on DGE-CEST MRI at clinical field strength (3 T). However, the detected DGE in brain tumors was found to be weak (on average < 1%) [4, 12]. In addition, 3-T scanning comes with challenges including the difficulty of separating DGE from direct water saturation and a low signal-to-noise ratio (SNR). This low SNR is challenging for dynamic experiments with long scan periods, which are inherently prone to motion artifacts [13]. To improve the detection of DGE, the use of several frequency offsets has been studied (*e.g.*, 1.2 ppm *versus* 2 ppm [14]). Other strategies to improve SNR in CEST imaging have been introduced, such as using truncated multilinear singular value decomposition denoising in pre-clinical use of DGE CEST [15], dynamic *B*_0_ correction [4], or principal component analysis (PCA) applied in amide proton transfer (APT) weighted CEST [16, 17]. However, dynamic *B*_0_ correction and PCA have not been combined in an attempt to improve DGE CEST MRI.

The objective of the current study was to develop an acquisition and analysis pipeline for DGE CEST on a hybrid 3-T PET/MRI system. This hybrid system was chosen to be able to directly compare the DGE-CEST signal with 18F-fluoro-deoxy-D-glucose uptake in future studies. We performed phantom, healthy volunteer, and patient studies to develop a novel approach of acquiring and analyzing DGE CEST MRI. We included the acquisition of static pre- and postinjection full Z-spectra and dynamically alternating two different offsets (1.2 and 2 ppm) to allow for dynamic *B*_0_ correction. During postprocessing, we applied PCA to improve DGE detection *in vivo*. Four patients diagnosed with brain metastases were included in this study to demonstrate the feasibility of our approach to detect DGE contrast in brain tumors.

## Methods

The study was conducted in compliance with the declaration of Helsinki and under the approval of the institutional ethics committee of the Erasmus MC, Rotterdam, The Netherlands (MEC-2020-0752). All scans were conducted using a 3-T hybrid PET/MRI scanner with a 24-channel head coil (Signa, General Electric, Chicago, USA).

### Phantom study

To optimize the local MRI acquisitions [18] for glucoCEST on the PET/MRI scanner, we first performed a phantom study. For this purpose, we created a phantom with varying concentrations of glucose and cross-linked bovine serum albumin, similar to the phantom used by Xu et al. [10]. Parameters that were optimized included the number of saturation pulses and frequencies used. This experiment resulted in the following acquisition parameters for the CEST scans, performed with a pulsed three-dimensional (3D) CEST sequence: root mean square B_1_ power of 1.5 µT; 120 Gaussian-shaped saturation pulses (saturation time 20 ms); 50% duty cycle; 35 frequency off-sets (∆ω) at ± 100, ± 50, ± 10, ± 8, ± 6, ± 5 ppm, from ± 4 to ± 1.5 in steps of 0.5 ppm, at ± 1.2, ± 1, ± 0.8, ± 0.5, ± 0.25, 0 ppm), and 4 images at 300 ppm as the reference (the last image was selected as the *S*_0_ image). A snapshot read-out of k-space was used as previously introduced by Deshmane et al. [19] but designed for MRI systems from General Electric and previously introduced by our group [18]. Additionally, the field of view was 220 × 180 × 42 mm^3^, matrix size 128 × 104 × 14, resolution 1.7 × 1.7 × 3.0 mm^3^, number of slices 14, flip angle 6°, acceleration factor 4, repetition time 7 ms, and echo time 3.2 ms. The acquisition time per offset was 7.2 s, yielding a scan time of 4:50 min:s.

### Participants

In total, ten healthy volunteers (median age 23 years, range 19−31 years) and four patients with brain metastases were included (Table 1). Written informed consent was obtained before any study procedures. The main inclusion criteria for healthy volunteers were age ≥ 18 years and no history of disease requiring prescription medication. For patients, aged ≥ 18 years, the presence of at least one enhancing brain tumor (either primary or secondary) and a World Health Organization performance status of 0 or 1 were required for study participation. The main exclusion criterion was diabetes mellitus type 1 or 2, for which venous blood glucose was checked at baseline (non-fasting, < 11.1 mmol/L) and prior to scanning (fasting, < 7.0 mmol/L). Additional inclusion and exclusion criteria are given in the Supplemental material (Table S1). All participants were required to fast for at least 8 h prior to the scan. On the day of the scan, each participant had an intravenous cannula placed in the arm for the administration of glucose, and an additional cannula in the contralateral arm to obtain venous blood samples. A bolus injection of 25 g of glucose (Glucose 50%, Braun®, Oss, NL) was administered with an automated power injector at an infusion rate of 0.25 mL/s (total infusion time of 200 s) to achieve a brief hyperglycemic state. Blood samples were taken just before and 1, 3, 5, 10, 20, 30, and 60 min after bolus infusion. The blood samples were collected in ethylenediaminetetraacetic acid tubes (2.0 mL, BD Vacutainer®, Vianen, NL) and immediately analyzed using a bedside glucometer (Accu-Chek®, Roche Diabetes Care Nederland, Almere, NL). To limit blood clotting, the cannulae were flushed with 0.9% NaCl immediately after each blood draw. To ensure a fresh sample, the first few drops of blood were discarded at each collection. Prior to, and at 30 and 60 min after infusion, blood samples were collected to measure insulin levels in healthy volunteers (6.0 mL, SST™ II Advance, BD Vacutainer®). All participants were monitored for at least 30 min after bolus infusion. At 5 days after scanning, a follow-up phone call was made to record adverse events.

**Table 1:** Characteristics of participants

ID	Sex	Age	Health status	Baseline (fasting) blood glucose level (mmol/L)	Peak blood glucose level (mmol/L)	Time after bolus infusion (min)	Peak insulin blood level (U/L)
1	M	25	Healthy volunteer	4.2	9.0	10	97
2	F	24	Healthy volunteer	4.7	11.3	5	93
3	F	19	Healthy volunteer	9.4^a^	11.2	1	217
4	F	24	Healthy volunteer	3.4	19.1	5	NA
5	F	24	Healthy volunteer	4.3	14.4	5	46
6	F	20	Healthy volunteer	3.2	9.8	30	137
7	M	20	Healthy volunteer	4.1	16.5	3	78
8	F	20	Healthy volunteer	6.7	11.7	10	122
9	F	25	Healthy volunteer	3.4	NA	NA	NA
10	M	31	Healthy volunteer	5.0	16.8	5	597
11	F	55	Brain metastasis (melanoma)	5.1	15.8	3	NA
12	F	70	Brain metastasis (non-small cell lung carcinoma)	2.7	13.9	5	NA
13	M	72	Brain metastasis (non-small cell lung carcinoma)	8.7^a^	18.6	20	NA
14	F	55	Brain metastasis (melanoma)	6.1	NA	NA	NA

*NA* Not available due to difficulty with the blood draw

^a^Likely a nonfasting result due to noncompliance to the fasting protocol

### Data acquisition

**Fig. 1:**
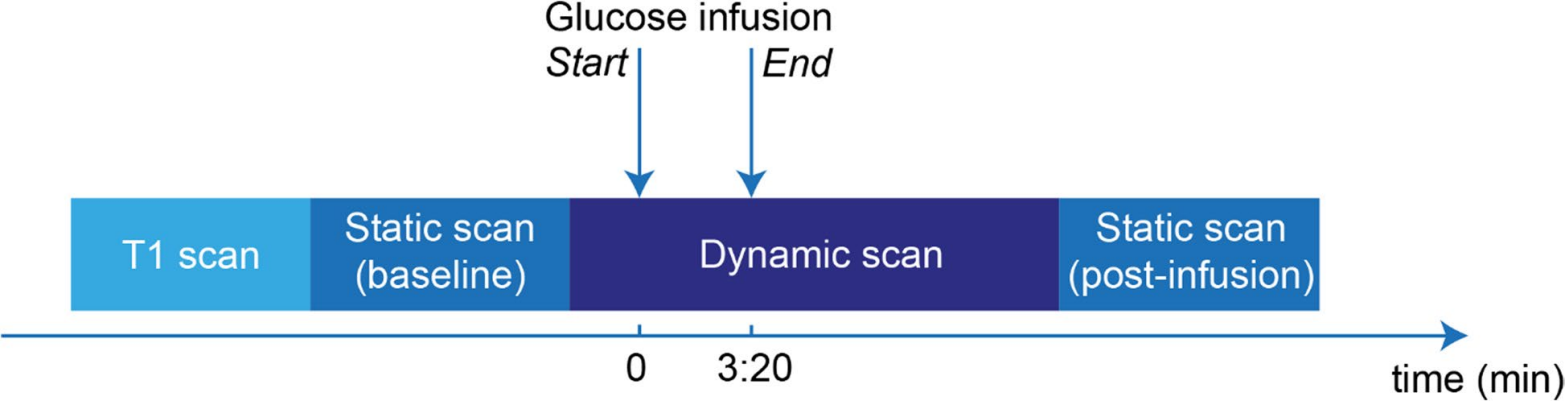
Data acquisition pipeline. Each volunteer was scanned statically by acquisition of a CEST sequence with a full Z-spectrum. Between the static scans, dynamic CEST imaging was obtained at two offsets (1.2 and 2.0 ppm). During the dynamic scan, glucose (50 mL 50% D-glucose [25 g] in 3:20 min:s) was administered, and venous blood samples were collected. *CEST* Chemical exchange saturation transfer

Each participant underwent two static scans (baseline scan and postinfusion scan: glucoCEST), as well as dynamic scanning during the glucose infusion (Fig. 1). For the static scan, we used the optimized sequence parameters from the phantom study to acquire the full Z-spectrum. The postinfusion static scan was performed 13 min after the start of infusion for the healthy volunteers.

The DGE scan was acquired for 128 time points (16 min) in healthy volunteers and 88 time points (11 min) in patients. In the first 5 volunteers, we performed single offset dynamic scans at 1.2 ppm (4 healthy volunteers) and 2 ppm (1 healthy volunteer). A more time-efficient acquisition for within-subject comparison between the two offsets was chosen for the final data sets acquired: interleaved dynamic scans with offsets of 1.2 and 2 ppm (9, including 4 patients). This approach additionally allowed for dynamic *B*_0_-correction during postprocessing. At the start of all dynamic scans, four images were acquired at 300 ppm to obtain the reference (*S*_0_) image for normalization in the analysis.

A high-resolution T_1_-weighted structural image was acquired for anatomical reference with a 3D inversion-recovery fast spoiled gradient-echo sequence (repetition time 6.1 ms, echo time 2.1 ms, voxel size 1.0 × 1.0 × 0.5 mm^3^, field of view 256 mm, 352 slices). In patients, images were also acquired after injection of Gadovist (Bayer, Leverkusen, Germany), a gadolinium-based contrast agent, at the dose of 7.5 mL, as part of the clinical protocol.

### Region of interest (ROI) definition

In healthy volunteers, whole brain grey matter (GM) and white matter (WM), cerebrospinal fluid (CSF) and the sagittal sinus (SS) were chosen as ROIs. GM/WM/CSF segmentation was performed automatically by FAST in FSL v5.0 [20] based on the brain-extracted T1-weighted structural images (BET in FSL). For the CSF ROI, the lateral ventricles were manually selected based on the automatically segmented CSF. The CEST image (S image) at 6 ppm was linearly registered to the T1-weighted image, resulting in transformation matrices from CEST to T1 space. These matrices were inverted and used to transform the ROI based on structural images into the CEST image acquired at 6 ppm from the static scan, and then to the second S image at 1.2 ppm from the dynamic scan. All alignments were linear registrations performed with FLIRT [21] within FSL. The SS was manually segmented based on the time series of DGE(t) at 2 ppm from the dynamic scan. The mean value of DGE during each time block for each ROI in each participant was calculated. Per ROI, a paired *t* test was performed to compare the group averaged glucoCEST from each static scan and to assess glucoCEST at 1.2 ppm *versus* 2 ppm in healthy volunteers.

### CEST data processing

Image analysis was done using in-house written MATLAB scripts (R2021a, The MathWorks, Natick, USA) [22] and the freely available functional MRI of the brain, FMRIB, software library (FSL 5.0, Oxford, UK) [21, 23]. The scripts are available upon request to the corresponding author. For the static scans, all the S images were aligned to the S image at 6 ppm with linear registration (FLIRT in FSL v5.0) for motion correction. The image at 6 ppm from the static sequences was then aligned to the first image acquired at 1.2 ppm from the dynamic scan. After registration, the Z-spectra and *B*_0_ field inhomogeneity correction were calculated in the same way as previously used by Wu et al. [18]: voxel-by-voxel shifts in *B*_0_ were calculated by first performing multi-pool Lorentzian fitting of the direct saturation and magnetization transfer effects to the Z-spectrum offsets between -1 ppm and 1 ppm and < -10 ppm and > 10 ppm. The resulting Lorentzian fit was calculated for the whole frequency range with a resolution of 1 Hz (0.0078 ppm) [24]. The offset with the minimum of the Lorentzian fit was set as the voxel *B*_0_ shift. The *B*_0_ shift map was saved for the baseline and postinfusion scans. *B*_0_ corrected *Z*-spectra were fitted by two-pool Lorentzian fitting as previously described [18], from which the amplitude of direct water saturation and magnetization transfer was obtained to evaluate the effects that influence the measurement of glucoCEST. B_0_ corrected Z-spectra, the amplitude of direct water saturation and magnetization transfer were averaged within the ROIs. To calculate the glucoCEST of each participant we used:

1 $$glu\mathrm{coCEST}={Z}_{\mathrm{ROI},\mathrm{ baseline}}\left(1.2\mathrm{ ppm}\right)-{Z}_{\mathrm{ROI},\mathrm{ post}}\left(1.2\mathrm{ ppm}\right)$$

For the dynamic scans, motion correction was performed in which all images were aligned to the second saturated image with mcflirt in FSL v5.0 [21]. Then, normalization was done by dividing all images by *S*_0_. After that, *S*/*S*_0_ as a function of time was divided into two series based on the frequency offset 1.2 or 2 ppm. Voxel-wise dynamic B_0_ correction was performed according to the study by Windschuh et al. [25]. In each voxel, a linear interpolation between *B*_0_ shift at the beginning (baseline) and the end (postinfusion) was performed. This resulted in a time series of 64 *B*_0_ shift maps. To obtain the corrected S/S_0_ at 1.2 or 2 ppm in each time point of the dynamic series an extrapolator was applied to fit the* Z*-spectra using two consecutive *S*/*S*_0_ (1.2 ppm) and *S*/*S*_0_ (2 ppm) images and the corresponding, interpolated *B*_0_ shift. We used the first three components of PCA performed on the thus corrected dynamic series as an empirical trade-off between removing noise whilst maintaining information on the glucose injection.

To compare the glucose enhancement in different time blocks, we computed DGE images at each time point and averaged the DGE over four-time blocks. The DGE from each time point was calculated based on Eq. 2:

2 $$\mathrm{DGE}(t)={(S}_{\mathrm{baseline}}-S(t))/{S}_{0}$$

where *S*_baseline_ was the mean *S* in block 1. The four-time blocks were defined as follows, where *t* = 0 equals the start of the infusion: block 1, baseline before the infusion; block 2, 0−3:20 min:s (during infusion); block 3, 3:20−6:40 min:s (first block after infusion); block 4, 6:40−10 min:s (second block after infusion).

### Statistical analysis

All statistical analysis was performed by SPSS (Released 2021. IBM SPSS Statistics for Windows, Version 28.0.1.0, Armonk, NY: IBM Corp.). Paired *t* tests were performed to compare the group averaged DGE per ROI per time block from dynamic scans before and after advanced correction (dynamic *B*_0_ and PCA) in healthy volunteers. For both static and dynamic scans, one sample *t* tests were performed to test whether the group averaged glucoCEST/DGE of each ROI were significantly different from 0. We considered *p* < 0.05 as significant.

### Patient study

In the patient study, the tumor ROI was the enhancing part of the tumor, manually delineated on the postcontrast T1-weighted image, which was first registered to the dynamic CEST scan. For the analysis of the patients, the WM ROI was generated in the same way as in the healthy volunteers, but only an ROI of approximately equal size as and contralateral to the tumor ROI was used as a reference region. Due to the location of the metastasis in the second patient, a region containing both white and grey matter in the contralateral cerebellum was manually selected on the dynamic CEST images as an ROI of healthy tissue.

DGE maps were calculated for each block to visually inspect the impact of motion correction and denoising on the images. The ROIs were used for the denoised time courses for DGE. No statistical analyses were done in the patient study, due to the small number of patients and heterogeneous results.

## Results

### Blood glucose levels

**Fig. 2:**
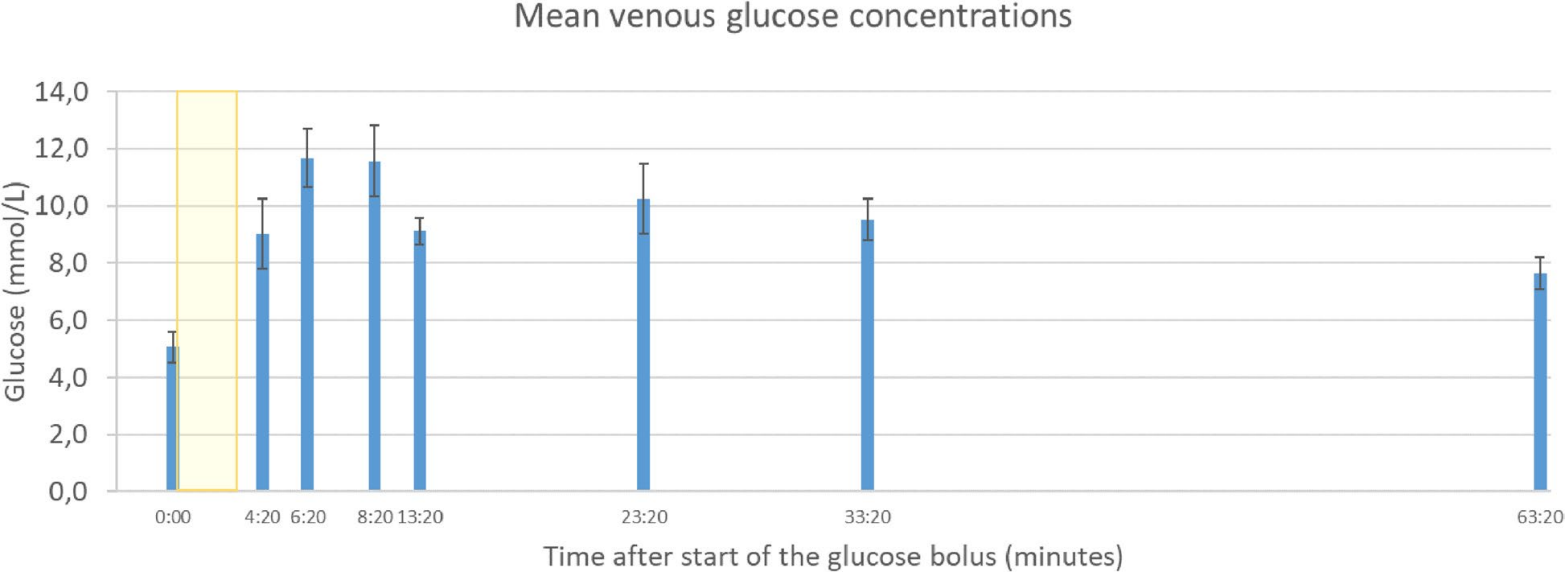
Mean changes in venous glucose concentrations over time (*n* = 14). The error bars indicate standard deviations. The yellow block indicates the infusion period of the glucose bolus

All participants had fasting levels of glucose < 7 mM/L, except for one healthy volunteer (Table 1, number 3) who had a prebolus fasting glucose level of 9.4 mM/L; she had probably not adhered to the 8-h fasting protocol, as there were no other signs or symptoms of diabetes mellitus. None of the participants experienced any acute infusion-related problems. One healthy volunteer (Table 1, number 1) reported signs of phlebitis (stiffness, erythema) at the site of glucose administration, starting one day after the scan. This was self-limiting, and resolved within 5 days. All participants showed an increase in venous blood glucose concentration following the glucose injection, with a median peak level at 5 min after the start of infusion. Following the first peak, glucose levels plateaued or showed a second, smaller peak between 10 and 30 min, after which the levels normalized (Fig. 2). During the static postinfusion scan, the venous blood glucose concentration was higher (*p* < 0.001) compared to baseline (Table 1). Postbolus venous blood insulin levels varied greatly between participants, with a median peak concentration of 109.5 U/L (range 46−597 U/L).

### Static scanning: glucoCEST in healthy volunteers

**Fig. 3:**
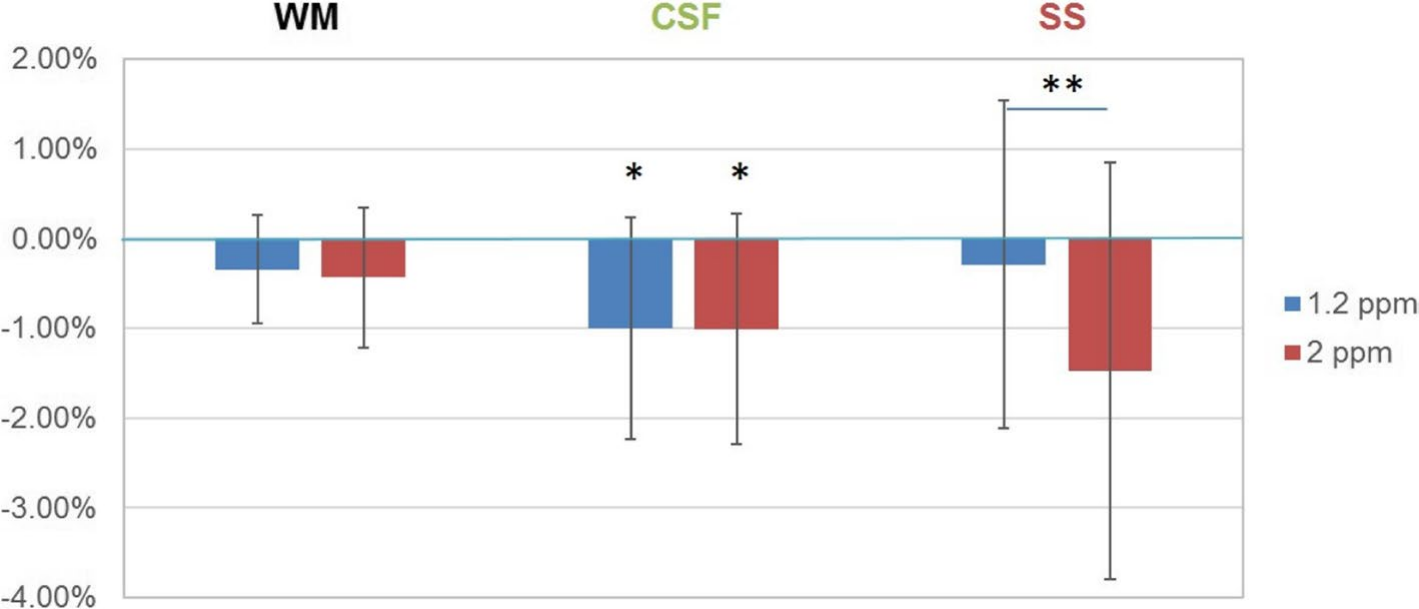
Mean glucoCEST in different regions of interest with standard deviations as error bars. *indicates a significant difference compared with 0 under one sample *t* test (*n* = 10, CSF at 1.2 ppm *p* = 0.039, CSF at 2 ppm *p* = 0.043). ** indicates significant difference in mean glucoCEST between 1.2 and 2 ppm (*n* = 10, *p* = 0.014). *CEST* Chemical exchange saturation transfer

In healthy volunteers, the average glucoCEST, calculated from the difference between the static baseline and postinfusion scans, was negative in GM, WM, CSF, and SS (Fig. 3, Table 2), albeit only significantly different from 0 in the CSF at 1.2 ppm (*p* = 0.039, one sample *t* test, *N* = 10) and at 2 ppm (*p* = 0.043, one sample *t* test, *N* = 10). The absolute glucoCEST at 2 ppm was higher than glucoCEST at 1.2 ppm for all three ROIs. However, this difference was only significant in the SS (*p* = 0.014, paired *t* test, *N* = 10).

**Table 2:** Group mean and standard deviation of glucoCEST/DGE, difference of magnetization transfer effect and *B*_0_ shift for different regions of interest in the brain

**Static**				
	*White matter*	*Grey matter*	*Cerebrospinal fluid*	*Sagittal sinus*
glucoCEST (@1.2 ppm)	-0.34% ± 0.60%	-0.73% ± 0.98%	-0.99% ± 1.24%	-0.29% ± 1.83%
glucoCEST (@2 ppm)	-0.43% ± 0.78%	-0.80% ± 0.11%	-1.01% ± 1.28%	-1.48% ± 2.32%
δmagnetization transfer^a^	-0.46% ± 0.88%	-0.29% ± 0.71%	-0.62% ± 1.42%	-0.39% ± 2.59%
δB0 shift (10^−2^ ppm) ^a^	-1.84 ± 4.07	-2.88 ± 3.54	-1.44 ± 3.93	-2.00 ± 8.75
**Dynamic**				
DGE (@2 ppm)	*White matter*	*Grey matter*	*Cerebrospinal fluid*	*Sagittal sinus*
DGEblock2, before	-0.10% ± 0.06%	-0.10% ± 0.07%	-0.10% ± 0.11%	-1.29% ± 1.10%
DGEblock2, after	-0.01% ± 0.14%	-0.09% ± 0.06%	-0.01% ± 0.19%	-1.11% ± 1.00%
DGEblock3, before	-0.14% ± 0.12%	-0.24% ± 0.03%	-0.18% ± 0.17%	-2.67% ± 1.57%
DGEblock3, after	-0.07% ± 0.21%	-0.24% ± 0.02%	-0.10% ± 0.34%	-2.44% ± 1.55%
DGEblock4, before	-0.05% ± 0.09%	-0.13% ± 0.05%	-0.14% ± 0.27%	-2.61% ± 1.85%
DGEblock4, after	-0.05% ± 0.10%	-0.16% ± 0.02%	-0.10% ± 0.22%	-2.50% ± 1.66%

*CEST* Chemical exchange saturation transfer, *DGE* Dynamic glucose-enhanced

^a^The difference of the magnetization transfer effect and B_0_ shift was calculated by using the value from the static postinfusion scan minus that from the static baseline scan

### Dynamic scanning: DGE in healthy volunteers

**Fig. 4:**
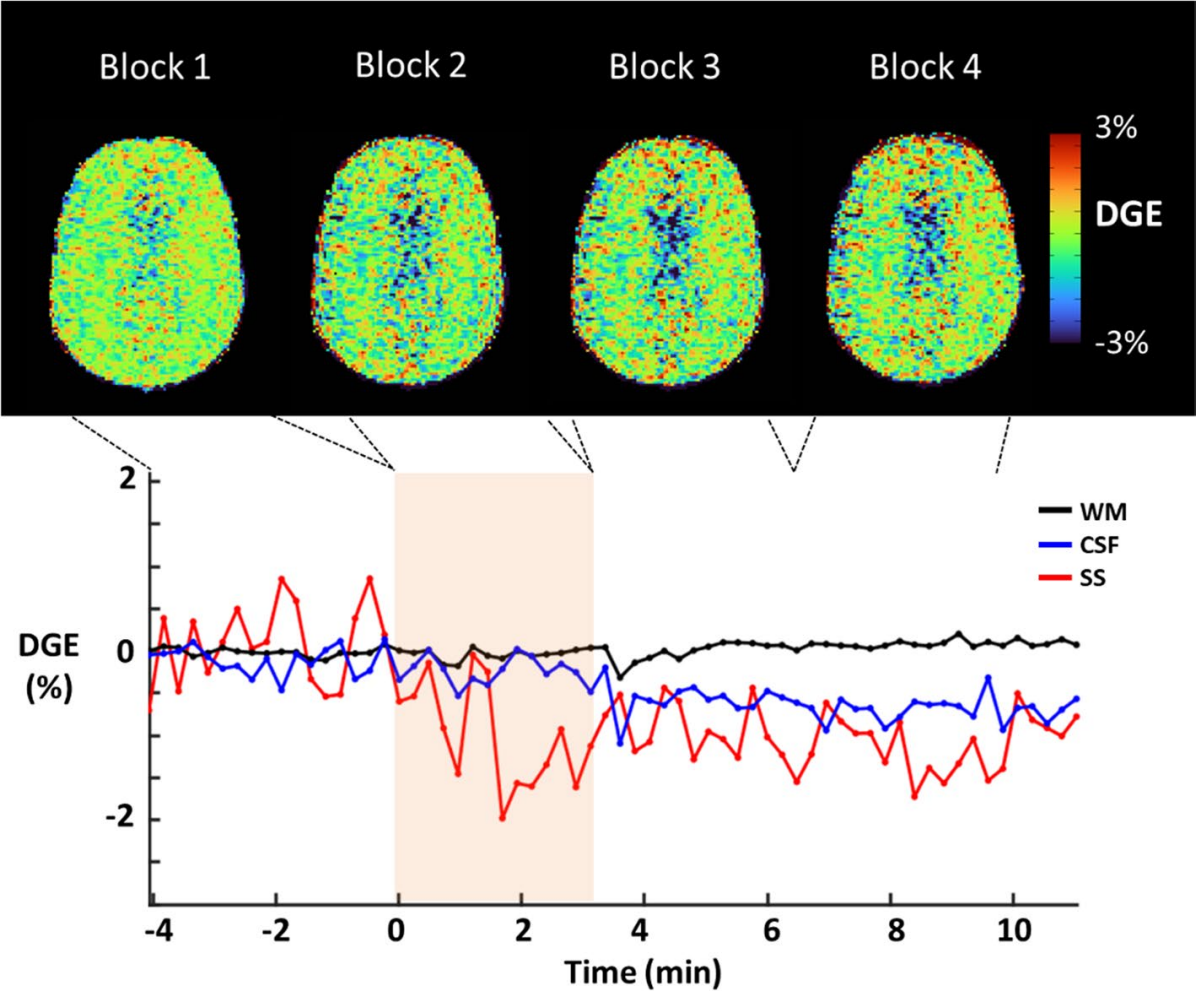
*Top*: averaged DGE in four-time blocks from one representative volunteer in different regions of interest (ROIs) before (block 1), during (block 2), and after (blocks 3 and 4) glucose infusion. *Bottom*: DGE changes over time in different ROIs from the same volunteer. The colored frame in the bottom time course indicates the time of glucose infusion. *CSF* Cerebrospinal fluid, *DGE* Dynamic glucose-enhanced, *ROIs* Regions of interest, *SS* Sagittal sinus, *WM* White matter

**Fig. 5:**
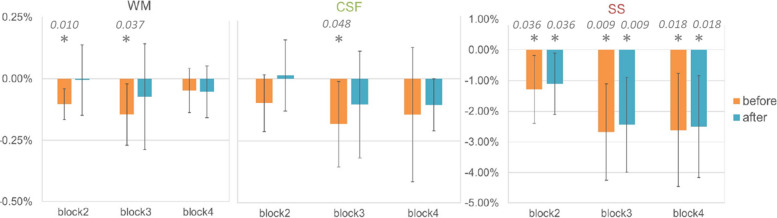
Mean DGE in different ROIs of the brain in time blocks, before (orange) and after (blue) advanced correction (dynamic *B*_0_ and principal component analysis). Error bars indicate standard deviation. Time is given from the start of the infusion: block 1, baseline before the infusion; block 2, 0−3:20 min:s (during infusion); block 3, 3:20−6:40 min:s; block 4, 6:40−10 min:s. * Indicates significantly different from 0 with one sample *t* test (*p* < 0.05, these *p*-values are stated in the figure), WM before correction, block 1 and 2, *p* = 0.010 and *p* = 0.037). *DGE* Dynamic glucose-enhanced

Figure 4 shows the time course of DGE in different ROIs from one representative volunteer. DGE could be detected during and after the glucose infusion. The DGE (group mean ± standard deviation, *N* = 6) at 2 ppm after advanced correction in WM (-0.01% ± 0.14%), GM (-0.09% ± 0.06%), and the CSF (-0.01% ± 0.19%) in time block 2 had a lower effect size compared to the DGE in the SS (-1.11% ± 1.00%). The DGE in other time blocks is shown in Table 2. For PCA denoising, the first three principal components explained 99.7% of the variance of the dynamic signal at 2 ppm (group average, *N* = 6). Visual inspection of the image quality of the dynamic scans showed local improvements in the inclusion of motion correction, *B*_0_ correction, and PCA. Figure 5 shows group averaged (*N* = 6) DGE for each ROI per time block at 2 ppm, before and after correcting for B_0_ inhomogeneity and including PCA. Before the correction, significantly negative DGE was found in the WM (blocks 2 and 3, *p* = 0.010, and *p* = 0.037 respectively), the CSF (block 3, *p* = 0.048), and the SS (blocks 2, 3, and 4, *p* = 0.036, *p* = 0.009, and *p* = 0.018, respectively) with one sample *t* tests. After correction, only the SS showed significant DGE in all three-time blocks (*p* = 0.042, *p* = 0.012, and *p* = 0.014, respectively). Including dynamic *B*_0_ correction and PCA denoising did not significantly change the group averaged DGE per time block in any ROI (paired *t*-tests, all *p* > 0.05).

### Patient characteristics

The first patient (female, 55 years) had metastatic melanoma and multiple brain metastases for four years with ongoing partial tumor response during systemic treatment met *BRAF/MEK-*inhibitors. The second (female, 70 years) and third patient (male, 72 years) had a newly diagnosed, untreated, solitary brain metastasis from non-small cell lung cancer. The fourth patient (female, 55 years) had two brain metastases (primary: melanoma), one of which was located in the brainstem that had been previously irradiated.

### DGE in brain metastases

**Fig. 6:**
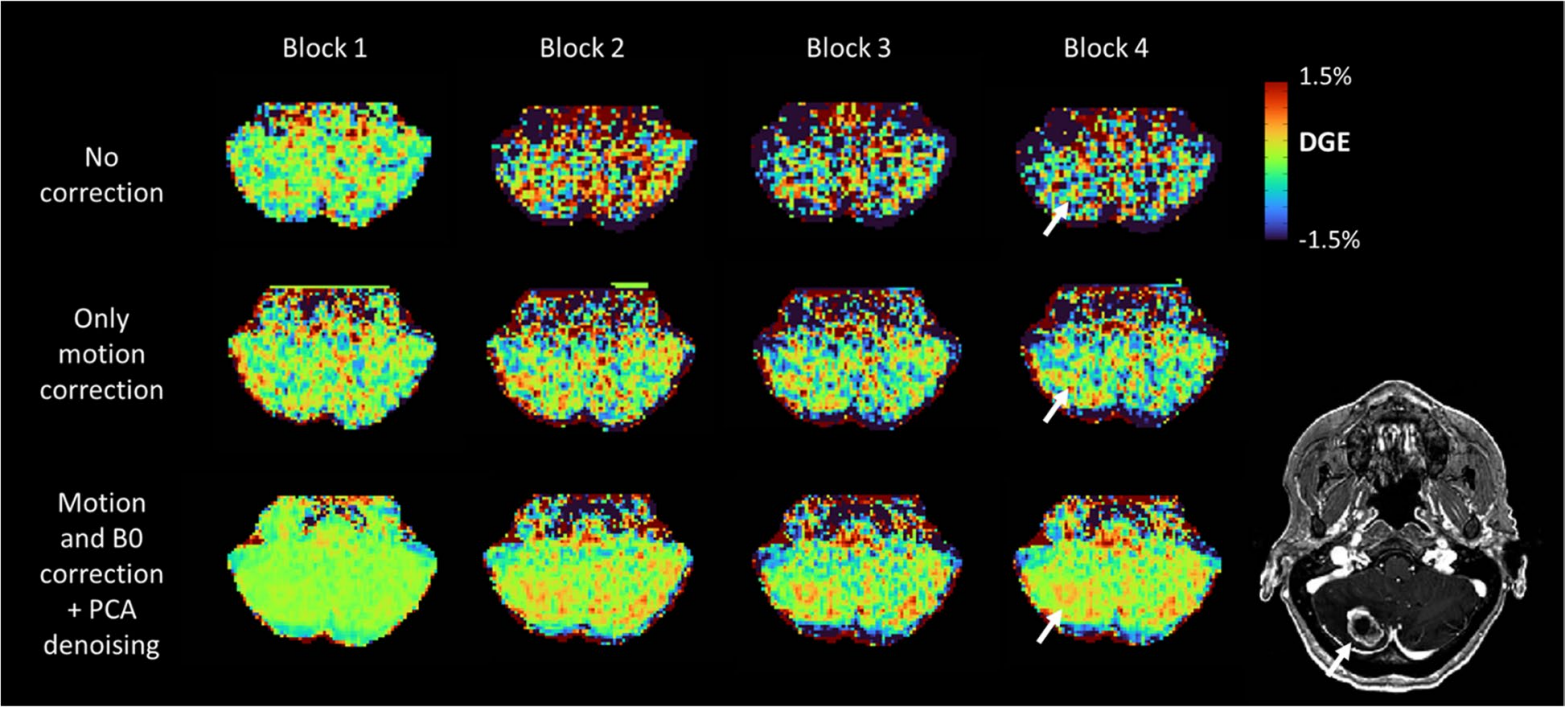
Example of DGE maps (2 ppm) for block 1 through 4 for patient 2, indicating the overall improvement of DGE map quality from no correction (top row), only motion correction (middle row), and with including *B*_0_ correction and principal component analysis denoising (bottom row). Axial slice through the cerebellum, containing enhancing tumor, as depicted in the axial view of the postcontrast T1-weighted image (bottom right). *DGE* Dynamic glucose-enhanced

**Fig. 7:**
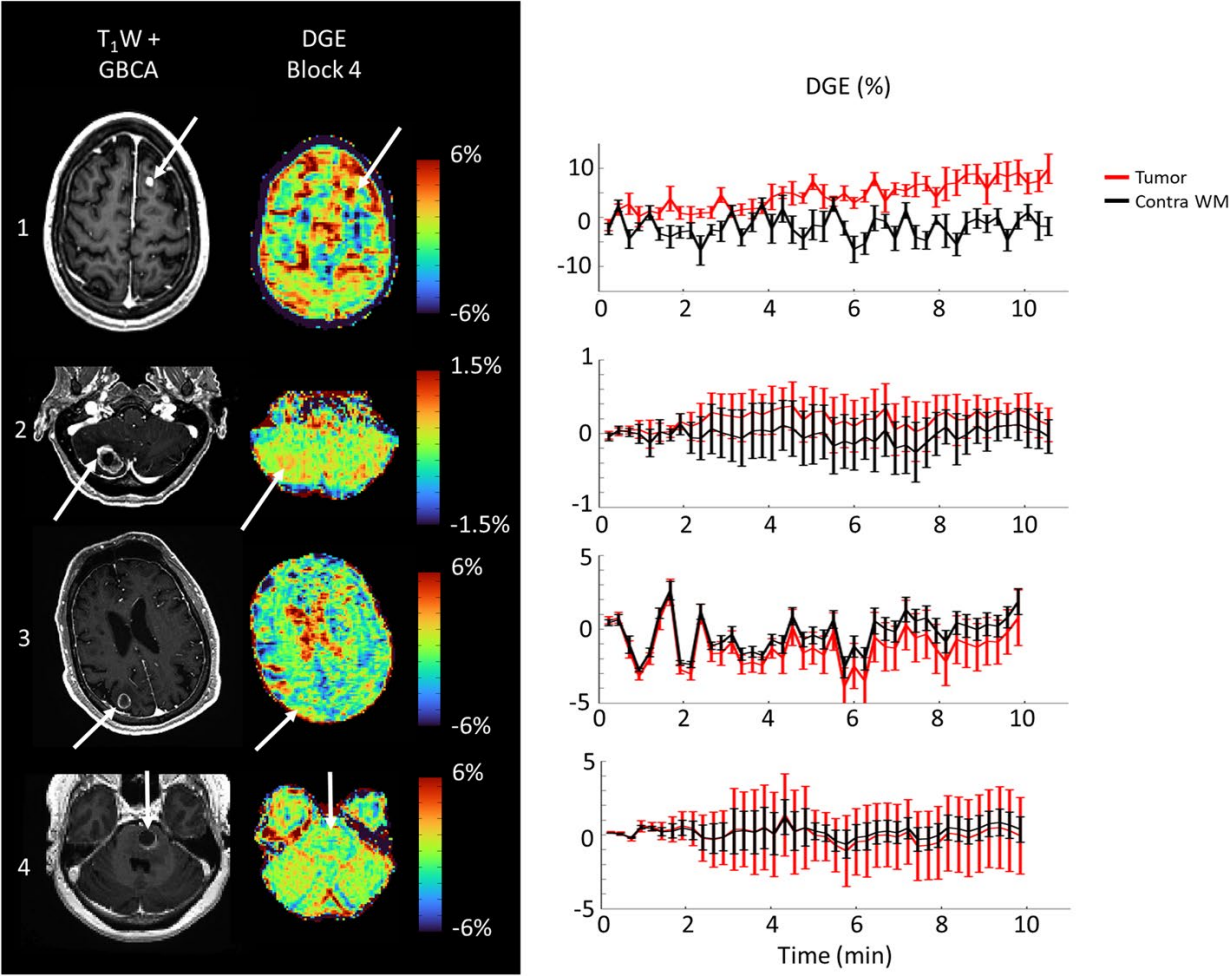
*Left:* postcontrast T1-weighted images and DGE maps for all four patients with brain metastases. DGE maps (2 ppm) are all corrected according to the full denoising postprocessing pipeline. *Right:* accompanying DGE time courses (2 ppm) for enhancing tumor and contra WM. Errorbars denote the standard deviation across the voxels within each ROI. *DGE* Dynamic glucose enhanced*, WM* White matter

In the patient data, the effect of advanced correction was similar to that in healthy volunteers, where additional correction for *B*_0_ inhomogeneity and PCA resulted in visually improved DGE images for all four patients (example in Fig. 6). The DGE contrast found between the tumor and the contralateral WM varied between patients. As can be seen in Fig. 7, for patients 1 and 2, DGE in the tumor was elevated compared to the contralateral WM, whereas limited contrast was found between the enhancing area of the tumor and contralateral WM for patients 3 and 4.

## Discussion

In the current study, we optimized DGE CEST MRI acquisition for both static and dynamic 3-T scanning, and developed a pipeline to process *in vivo* glucose-enhanced CEST data both in healthy brain parenchyma and in brain metastases. In healthy volunteers, we compared the detection of glucoCEST at 1.2 and 2 ppm, where 2 ppm resulted in a stronger signal. To overcome the low SNR of DGE *in vivo*, we included advanced correction (dynamic *B*_0_ correction and PCA-based denoising) in the processing pipeline. While no statistically significant effect of the advanced correction during postprocessing was found for healthy volunteers, the application of advanced correction visually improved the DGE maps in patients with brain metastases, leading to improved detection of DGE contrast between tumor and healthy tissue in two of four patients. Therefore, this work demonstrates feasibility of using 3-T DGE CEST MRI in patients with brain metastases.

In the static scans in healthy volunteers, the glucoCEST values in the SS region were negative and the absolute value was on average lower than the effect size of 2% at 2 ppm found at 10 mM in the phantom study. This finding might in part be explained by the lower increase of glucose concentrations in the venous blood of the volunteers (on average a 4-mM increase at the time of postinfusion scan, compared to the 10 mM in the phantom). GlucoCEST at 1.2 ppm is assumed to include more CEST effect of fast exchanging hydroxyl protons, but it is also more affected by direct water saturation and *B*_0_ inhomogeneity. A trend towards increased absolute glucoCEST at 2 ppm *in vivo*, in particular at later time blocks, may be a reflection of these effects and is in line with previous work [14].

In the dynamic scans in healthy volunteers, dynamic *B*_0_ correction recovered the DGE signal in the frontal lobe in line with previous work [4]. The PCA reduced noise in the DGE signal during the dynamic scan and provided a presumably better approximation of the true glucoCEST signal at 1.2/2 ppm [16]. This is reflected by nonsignificant DGE effects in WM and CSF after the advanced corrections, which is in line with previous studies in healthy volunteers [3, 14].

To the best of our knowledge, this is the first report on applying PCA to DGE CEST data. An alternative to using PCA for denoising in DGE experiments is presented by Huang et al. [15], who have recently applied truncated multilinear singular value decomposition to DGE CEST data from a mouse model of glioma. In contrast to our PCA analysis, which only included components in the time domain, the truncated multilinear singular value decomposition approach includes the spatiotemporal correlation of the dynamic imaging data. Huang et al. hereby showed good results in isolating the temporal DGE signal and improved contrast within an image at a single time point. Future work comparing the use of PCA to truncated multilinear singular value decomposition for denoising human DGE data is therefore warranted.

Within 10 min after the start of the glucose infusion, the absolute DGE values increased during the infusion period (time block 2), which corresponded to the increased glucose levels in venous blood. We did not observe a decrease in venous blood glucose in the delayed phase (7−10 min after start of infusion), in contrast to the study by Xu et al. [10], where the blood glucose levels decreased within 10 min after the glucose infusion. This difference may be explained by between-subject variability of the insulin response as previously demonstrated in glucose tolerance studies [26, 27]. Since the SNR of DGE is small, the variable blood glucose levels affect the DGE signal. To detect the largest DGE signal change postglucose infusion, monitoring of blood glucose concentrations is thus recommended for DGE.

Considering safety, we chose a glucose infusion duration of 3:20 min, in line with a recent study of Seidemo et al. [14]. They found that an infusion duration of 3–4 min is preferable in DGE experiments, to limit side effects such as thrombophlebitis while maintaining glucoCEST effect size. Still, one of our participants had clinical signs of phlebitis (not confirmed by ultrasound) with complete resolution of symptoms within 5 days. Note that this is similar as reported by Seidemo et al. as well as another DGE study [28].

We detected negative glucoCEST/DGE signal in the healthy tissue ROIs, which may be contrary to expectation based on expecting higher glucose concentrations in healthy tissue ROIs leading to decreased *Z-*spectra values at 1.2−2.0 ppm. However, negative glucoCEST/DGE in healthy tissue was also found in recent research [3, 14]. A potential cause could be concomitant changes in magnetization and direct transfer effects during and after infusion, which are not compensated for in our current calculations of glucoCEST/DGE.

In patients with brain metastases, acquisition of DGE at 2 ppm and applying dynamic *B*_0_ correction and PCA led to improved contrast between brain metastasis and healthy tissue in two of the four patients. In these two patients, a stronger DGE signal was observed compared to normal brain tissue in the same area as contrast enhancement. No differences between tumor and contralateral normal-appearing white matter were measured in the other two patients. These variable results are in line with other DGE 3-T studies that included patients with primary brain tumors; if an elevated DGE signal is found within the tumor region, it mostly overlaps with contrast enhancement, albeit not as a one-to-one match [3, 4, 12]. The increased DGE signal may thus reflect glucose leakage through a disrupted blood–brain barrier, which suggests that DGE imaging provides contrast in a similar way as the leakage of the contrast agent through a disrupted blood–brain barrier into the extravascular space [5, 6]. However, the cellular origin of DGE contrast is still unclear. Other effects related to elevated glucose concentrations, such as increases in arterial osmotic pressure [29] or potential changes in pH, magnetization transfer, or direct transfer effects, are not yet ruled out. Since previous studies did not include patients with brain metastases more research into the origin of DGE signal changes at 3 T for patients with brain metastases remains warranted.

This study has limitations. In our dynamic study, we were unable to detect arterial input functions. One potential reason could be the use of smaller voxel sizes as compared to previous work with arterial input functions [3, 10]. Smaller voxel sizes limit partial volume effects since there is less mixing of signals from several types of tissues within one voxel, which is important for mapping heterogeneous lesions such as tumors. On the other hand, it may have caused a loss of SNR resulting in reduced detection of arterial input functions in our data sets. Additionally, we used an empirical choice of the first three components of the PCA for all participants to calculate the DGE. This was done so based on the combination of the explained variance as well as improved signal-to-noise in the resulting DGE images, judged by visual inspection. Future work should investigate the optimal number of components, which may vary per participant, to determine the DGE as was done in previous work in which PCA was applied to APT-weighted CEST MRI [16, 17]. Finally, we did not use an additional acquisition for *B*_0_ correction (*e.g.*, WASSR or WASABI) of the Z-spectra before and after infusion due to time constraints on the imaging protocols. Future work including such separate acquisitions might improve the accuracy of the *B*_0_ fieldmaps, further improving the glucoCEST and DGE. This includes investigating the optimal design of the acquisition approach for the CEST sequence we used, as it allows for incorporating images acquired after RF saturation pulses with varying saturation powers and off-resonance frequencies.

In summary, we achieved improved detection of brain metastases with glucose-enhanced CEST MRI at 3 T by combining glucoCEST and dynamic DGE CEST together with applying dynamic B_0_ correction and PCA denoising. Our work demonstrates the feasibility and provides both the acquisition and analysis framework for future patient studies with glucose-enhanced CEST at clinical field strength.

### Supplementary Information

**Additional file 1: Table S1.** Inclusion and exclusion criteria for study participation.

## Data Availability

All data and scripts for analysis are available upon request to the corresponding author.

## Abbreviations

3D: Three-dimensional
APT: Amide proton transfer
CEST: Chemical exchange saturation transfer
CSF: Cerebrospinal fluid
DGE: Dynamic glucose-enhanced
FSL: Functional MRI of the brain software library
GM: Grey matter
MRI: Magnetic resonance imaging
PCA: Principal component analysis
PET: Positron-emission tomography
ROI: Region of interest
SNR: Signal-to-noise-ratio
SS: Sagittal sinus
WM: White matter

## References

[1] Warburg O, Wind F, Negelein E (1927). The metabolism of tumors in the body. J Gen Physiol.

[2] Wang J, Weygand J, Hwang K-P (2016). Magnetic resonance imaging of glucose uptake and metabolism in patients with head and neck cancer. Sci Rep.

[3] Xu X, Sehgal AA, Yadav NN (2020). D-glucose weighted chemical exchange saturation transfer (glucocest)-based dynamic glucose enhanced (dge) mri at 3t: early experience in healthy volunteers and brain tumor patients. Magn Reson Med.

[4] Herz K, Lindig T, Deshmane A (2019). T1ρ-based dynamic glucose-enhanced (dgeρ) mri at 3 t: Method development and early clinical experience in the human brain. Magn Reson Med.

[5] Chan KW, McMahon MT, Kato Y (2012). Natural d-glucose as a biodegradable mri contrast agent for detecting cancer. Magn Reson Med.

[6] Nasrallah FA, Pagès G, Kuchel PW, Golay X, Chuang KH (2013). Imaging brain deoxyglucose uptake and metabolism by glucocest mri. J Cereb Blood Flow Metab.

[7] Rivlin M, Horev J, Tsarfaty I, Navon G (2013). Molecular imaging of tumors and metastases using chemical exchange saturation transfer (CEST) MRI. Sci Rep.

[8] Walker-Samuel S, Ramasawmy R, Torrealdea F (2013). In vivo imaging of glucose uptake and metabolism in tumors. Nat Med.

[9] Xu X, Chan KWY, Knutsson L (2015). Dynamic glucose enhanced (dge) mri for combined imaging of blood–brain barrier break down and increased blood volume in brain cancer. Magn Reson Med.

[10] Xu X, Yadav NN, Knutsson L et al (2015) Dynamic glucose-enhanced (dge) mri: Translation to human scanning and first results in glioma patients. Tomography 1:105–14. 10.18383/j.tom.2015.00175. 10.18383/j.tom.2015.00175 PMC4710854 26779568

[11] Knutsson L, Xu X, van Zijl PCM, Chan KWY (2023). Imaging of sugar-based contrast agents using their hydroxyl proton exchange properties. NMR Biomed.

[12] Bender B, Herz K, Deshmane A et al (2021) Glint: glucocest in neoplastic tumors at 3 t—clinical results of glucocest in gliomas. MAGMA 10.1007/s10334-021-00982-5 10.1007/s10334-021-00982-5 PMC8901469 34890014

[13] Zaiss M, Herz K, Deshmane A (2019). Possible artifacts in dynamic cest mri due to motion and field alterations. J Magn Reson.

[14] Seidemo A, Lehmann PM, Rydhög A (2022). Towards robust glucose chemical exchange saturation transfer imaging in humans at 3 t: arterial input function measurements and the effects of infusion time. NMR Biomed.

[15] Huang J, Lai JHC, Han X (2022). Sensitivity schemes for dynamic glucose-enhanced magnetic resonance imaging to detect glucose uptake and clearance in mouse brain at 3 t. NMR Biomed.

[16] Breitling J, Deshmane A, Goerke S (2019). Adaptive denoising for chemical exchange saturation transfer mr imaging. NMR Biomed.

[17] Casagranda SP, C., Romdhane F, Firippi E et al (2022) Prinicipal component selections and filtering by spatial information criteria for mutli-acquisition cest mri denoising. Joint annual meeting ISMRM-ESMRMB. 2080

[18] Wu Y, Charles Wood T, Arzanforoosh F et al 3d apt and noe cest-mri of healthy volunteers and patients with non-enhancing glioma at 3 t. MAGMA 2022. 10.1007/s10334-021-00996-z 10.1007/s10334-021-00996-z PMC8901510 34994858

[19] Deshmane A, Zaiss M, Lindig T (2019). 3d gradient echo snapshot cest mri with low power saturation for human studies at 3t. Magn Reson Med.

[20] Zhang Y, Brady M, Smith S (2001). Segmentation of brain mr images through a hidden markov random field model and the expectation-maximization algorithm. IEEE Trans Med Imaging.

[21] Jenkinson M, Bannister P, Brady M, Smith S (2002). Improved optimization for the robust and accurate linear registration and motion correction of brain images. Neuroimage.

[22] Strupp JP (1996). Stimulate: A gui based fmri analysis software package. Neuroimage.

[23] Woolrich MW, Jbabdi S, Patenaude B (2009). Bayesian analysis of neuroimaging data in fsl. Neuroimage.

[24] Zhou J, Blakeley JO, Hua J (2008). Practical data acquisition method for human brain tumor amide proton transfer (apt) imaging. Magn Reson Med.

[25] Windschuh J, Zaiss M, Ehses P, Lee JS, Jerschow A, Regatte RR (2019). Assessment of frequency drift on cest mri and dynamic correction: application to gagcest at 7 t. Magn Reson Med.

[26] Chen MEI, Porte DJ (1976). The effect of rate and dose of glucose infusion on the acute insulin response in man. J Clin Endocrinol Metab.

[27] Nolfe G, Spreghini MR, Wietrzycowska Sforza R, Morino G, Manco M (2012). Beyond the morphology of the glucose curve following an oral glucose tolerance test in obese youth. Eur J Endocrinol.

[28] Kim M, Torrealdea F, Adeleke S et al (2019) Challenges in glucocest mr body imaging at 3 tesla. Quant Imaging Med Surg 9:1628–40. 10.21037/qims.2019.10.05 10.21037/qims.2019.10.05 PMC6828585 31728307

[29] Choi W, Chung JJ, Jin T, Kim SG (2017) Effect of osmolality on dynamic glucose enhanced(dge) mri. In: Proceedings of the International Society for Magnetic Resonance in Medicine, 25th Annual Meeting & Exhibition. 0194

