# Supplementary material for: Improved postprocessing of dynamic glucose-enhanced CEST MRI for imaging brain metastases at 3 T
Source: Eur Radiol Exp. 2023 Dec 8;7:78. doi: 10.1186/s41747-023-00390-5 (PMC10709288; doi:10.1186/s41747-023-00390-5)
Supplement: Supplementary file 1 — Additional file 1: Table S1. Inclusion and exclusion criteria for study participation. [file 41747_2023_390_MOESM1_ESM.docx]

**Improved postprocessing of dynamic glucose-enhanced CEST MRI for imaging brain metastases at 3 T**

**ELECTRONIC SUPPLEMENTARY MATERIAL**

**Table S1.** Inclusion and exclusion criteria for study participation.

| **Inclusion criteria** | **Exclusion criteria** |
| --- | --- |
| **For all subjects (healthy volunteers and patients)** | |
| Age ≥18 years | Diabetes mellitus type 1 or 2 (determined from medical history or 2 measurements on separate days of a non-fasting glucose level ≥11.1 mmol/L, or fasting level ≥7.0 mmol/L) |
| Baseline non-fasting venous blood glucose level <11.0 mmol/L | Blood iron deficiency (hemoglobin concentration <12 g/dL) |
| Signed and dated written informed consent prior to any study-specific procedures | History of seizures |
|  | MR-related exclusion criteria (e.g. metal implants, claustrophobia) |
|  | Somatic or psychiatric disease/condition or medication that may interfere with the study objectives and assessments |
| **For patients** | |
| WHO performance status 0 or 1 | Severe hepatic disease/liver transplant |
|  | Renal disease (from medical history or an estimated glomerular filtration rate [eGFR] <60mL/min) |
